# Site-Selective Ribosylation of Fluorescent Nucleobase Analogs Using Purine-Nucleoside Phosphorylase as a Catalyst: Effects of Point Mutations

**DOI:** 10.3390/molecules21010044

**Published:** 2015-12-28

**Authors:** Alicja Stachelska-Wierzchowska, Jacek Wierzchowski, Agnieszka Bzowska, Beata Wielgus-Kutrowska

**Affiliations:** 1 Department of Physics and Biophysics, University of Varmia & Masuria in Olsztyn, 4 Oczapowskiego St., 10-719 Olsztyn, Poland; jacek.wie@uwm.edu.pl; 2 Division of Biophysics, Institute of Experimental Physics, University of Warsaw, Zwirki i Wigury 93, 02-089 Warsaw, Poland; abzowska@biogeo.uw.edu.pl (A.B.); beata@biogeo.uw.edu.pl (B.W.-K.)

**Keywords:** fluorescent nucleosides, enzymatic ribosylation, 8-azapurines, purine nucleoside phosphorylase

## Abstract

Enzymatic ribosylation of fluorescent 8-azapurine derivatives, like 8-azaguanine and 2,6-diamino-8-azapurine, with purine-nucleoside phosphorylase (PNP) as a catalyst, leads to N9, N8, and N7-ribosides. The final proportion of the products may be modulated by point mutations in the enzyme active site. As an example, ribosylation of the latter substrate by wild-type calf PNP gives N7- and N8-ribosides, while the N243D mutant directs the ribosyl substitution at N9- and N7-positions. The same mutant allows synthesis of the fluorescent N7-β-d-ribosyl-8-azaguanine. The mutated form of the *E. coli* PNP, D204N, can be utilized to obtain non-typical ribosides of 8-azaadenine and 2,6-diamino-8-azapurine as well. The N7- and N8-ribosides of the 8-azapurines can be analytically useful, as illustrated by N7-β-d-ribosyl-2,6-diamino-8-azapurine, which is a good fluorogenic substrate for mammalian forms of PNP, including human blood PNP, while the N8-riboside is selective to the *E. coli* enzyme.

## 1. Introduction

Purine-nucleoside phosphorylase (PNP, E.C.2.4.2.1) is a key enzyme of purine metabolism, important, *inter alia*, for the proper activity of the immune system in mammals [1,2,3]. Potent inhibitors of PNP, like immucillin H (forodesine) and some analogs [4], are clinically approved to treat lymphomas [4,5,6], and others are considered as potential anti-parasitic and anti-malarial drugs [4,7]. Bacterial forms of PNP are of interest because they can be used as a suicidal-gene in cancer chemotherapy [8]. Besides this, PNP is used as a catalyst in the gram-scale preparative ribosylation of purines and purine analogs [9,10,11], thanks to the reverse (synthetic) pathway of the phosphorolytic process. Nucleoside analogues are widely applied as pharmaceuticals and as biochemical probes for enzymological studies [5,6,7,9,10,11,12,13].

**Figure 1:**
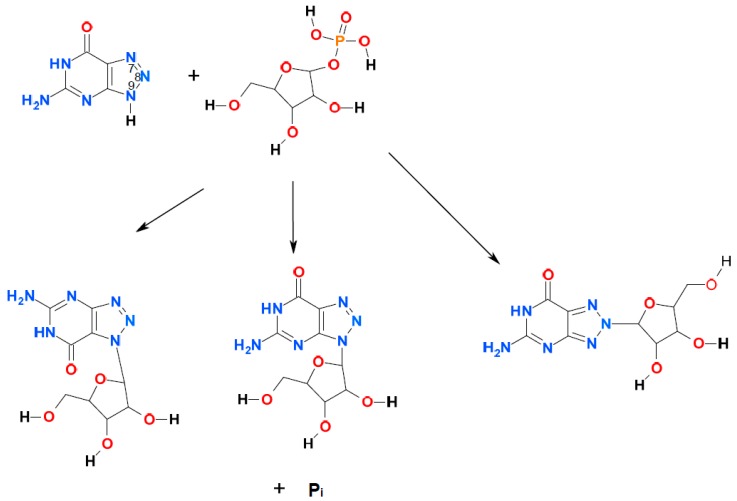
Ribosylation of 8-azaguanine with α-d-ribose-1-phosphate: possible reaction products. The purine numbering is maintained for simplicity. Only the major tautomeric form of 8-azaguanine (N(9)H) is shown.

In the preceding papers [14,15], we have demonstrated that enzymatic ribosylation of some 8-azapurines leads not only to the canonical nucleoside analogs, but also to non-typical, and highly fluorescent ribosides, ribosylated at the N7- and N8-positions (see Figure 1 below). Now we present a kinetic analysis of these processes, with application of several wild and mutated forms of PNP as catalysts. We will demonstrate a considerable selectivity of the 8-azapurine ribosylation sites with various PNP forms, and a remarkable sensitivity of the ribosylation process to point mutations at the critical active site residue. Finally, we present an example of potential analytical application of the obtained compounds to blood PNP determination.

## 2. Results and Discussion

In comparison with natural purines, their 8-aza analogues are not as good PNP substrates, but their ribosides are highly fluorescent and therefore can be utilized as probes in enzymology or clinical investigations [11]. Our aim was to identify those forms of PNP which can be used as catalyst in the effective and selective enzymatic syntheses of these ribosides.

We have investigated bacterial (*E. coli*) and mammalian (calf) forms of PNP, as the most widely accessible, and representing two main classes of the enzyme, as well as their mutated forms, which has been previously shown to express altered activity of the phosphorolytic process [1]. In particular, the N243D mutant of the calf and human enzymes is known to catalyze the phosphorolysis not only of Guo and Ino (as do the wild forms), but also of Ado [1,16]. We expected that the analogous mutant of the *E. coli* PNP, D204N, obtained recently [17], will be also interesting as a potential catalyst.

In the ribosylation reactions, we used α-d-ribose-1-phosphate, prepared enzymatically (see Section 3 for details), as a ribosyl donor [14]. Additionally, we have also measured kinetics of phosphorolysis of 8-azapurine ribosides in the phosphate buffer.

### 2.1. Ribosylation of 8-Azaguanine and 2,6-Diamino-8-azapurine

**Figure 2:**
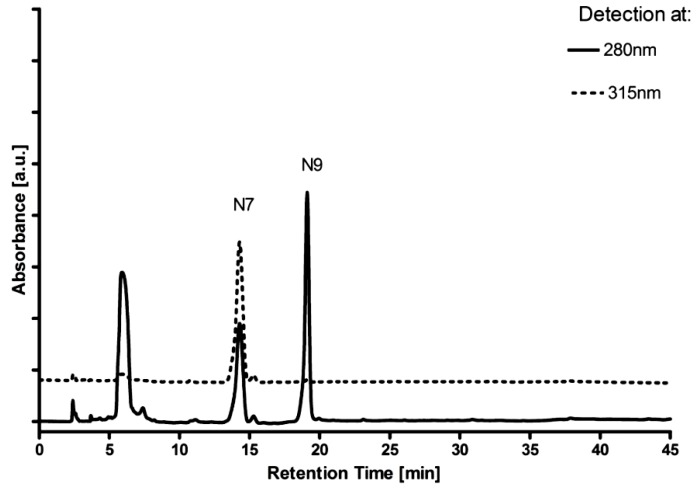
The HPLC elution profile for the mixture obtained from the ribosylation of 8-azaguanine with α-d-ribose-1-phosphate as a ribose donor, catalyzed by the N243D mutant of the calf PNP. The Kromasil C8 column (5 µm × 250 × 10 mm) was used, and the reaction mixture eluted with water (10 min) followed by 0%–30% methanol gradient. The first peak (~6 min retention time) was identified as unreacted 8-azaguanine.

Ribosylation of 8-azaguanine (8-azaGua) and 2,6-diamino-8-azapurine (8-azaDaPur) was followed spectrophotometrically, and the reaction products analyzed using HPLC separation coupled with spectrophotometric and fluorimetric detectors. The products were identified by comparing their spectral properties with those published earlier [18,19,20]. The non-typical ribosides of both substrates are spectrally distinct from the N9-nucleosides in that the UV spectra of the formers are quite red-shifted, allowing relatively easy detection at 315 nm. Typical elution profile from the C8-column is shown on Figure 2.

As pointed earlier, enzymatic ribosylation of 8-azaguanine goes fairly rapidly, when catalyzed by calf PNP [11], and the only product of this process is N9-riboside. By contrast, the mutated (N243D) form of the enzyme gives as a major product N7-riboside, although the overall rate of this process is quite slow. Similar qualitative differences can be observed for 8-azaDaPur ribosylation: while the wild-type enzyme gives a mixture of N8 and N7 forms, the application of the N243D mutant gives mainly N7, some N9- and virtually no N8-riboside (see Table 1).

**Table 1:** Kinetic parameters for enzymatic ribosylation of selected 8-azapurines in 25 mM HEPES buffer, pH 6.6, by α-d-ribose-1-phosphate, using various forms of PNP (nd = not determined). Standard errors are estimated to be ~15%.

*Substrate/Enzyme*	*K_m_ (µM)*	V_max_ (Relative) *	*Approximate Product Ratio: N9-riboside: N8-riboside:N7-riboside*
8-azaGua/calf PNP-wt	~90	21	1:0:0
8-azaGua/calf PNP-N243D	>100	>0.2 ***	1:0:2
8-azaGua/*E. coli* PNP-wt	>200	~1 ***	20:1:0
8-azaGua/*E. coli* PNP-D204N	nd	traces	predominantly N9
8-azaDaPur/calf PNP-wt	60	~1	0:1:1 **
8-azaDaPur/calf PNP-N243D	35	0.6	1:0:3
8-azaDaPur/*E. coli* PNP-wt	>200	~3 ***	1:2:0
8-azaDaPur/*E. coli* PNP-D204N	>200	~0.4 ***	10:1:0

* relative to guanine ribosylation under the same conditions (=100); ** this ratio is dependent on the reaction progress; *** reaction rates measured at 200 µM substrate.

The *E. coli* PNP (wild type) gives predominantly N9-riboside of 8-azaguanine, with some participation of a highly fluorescent N8-form. Similar specificity was obtained using the D204N mutant, although the reaction was much slower. With 8-azaDaPur as a substrate, the N8-riboside becomes a dominant ribosylation product for the wild-type enzyme, while the mutant gave mainly N9-nucleoside. Ribosylation rates are typically lower for the mutated enzymes, comparing with the wild types. The K_m_ values, determined by standard methods, are lower for the calf enzymes (Table 1), while the *E. coli* PNP was not saturated under the applied conditions.

The *E. coli* PNP is known to ribosylate adenine and 8-azaadenine [1,11]. In both cases, the N9-ribosides were the only detectable ribosylation products. We have found that the D204N mutant reacting with the latter substrate does produce also a non-typical product with red-shifted UV spectra, tentatively identified as N8-riboside (data not shown).

The altered specificity of the PNP mutants towards some of the purine analogs has been observed in other laboratories [21]. It can be related to differences in binding modes of 8-azapurine bases in the PNP active site, including binding in the “upside-down” position (rotation by 180 deg when compared with standard binding mode). In fact, such an “upside-down” binding mode was already observed in the crystal structure of calf PNP complexed with N7-acycloguanosine inhibitor [22]. The altered binding geometry can possibly be associated also with prototropic tautomerism or proton affinity alterations [23,24,25,26]. This illustrates remarkable plasticity of the mammalian and bacterial PNP active site, which may be profited from in designing new more potent and better membrane-permeable PNP inhibitors—potential pharmaceutical agents [1,2,3,4,5,6,7].

There is a considerable progress in enzymatic synthesis of nucleoside analogues on a gram scale using PNP as a catalyst [9,10,27,28,29,30]. This work demonstrates that in some substrates there is a possibility to alter ribosylation specificity of PNP by point mutations, an effect noted also by other authors [21]. Although the non-typical ribosides are less biologically active than their N9-β isomers, they sometimes exhibit interesting spectral properties and could be applied as fluorescent probes [14,15,21].

### 2.2. Phosphorolysis of 8-Azaguanine and 2,6-Diamino-8-azapurine Ribosides

On the basis of the micro-reversibility principle, it can be expected that mutations in the active site of PNP can alter the phosphorolytic processes as well. We have therefore purified the 8-azapurine ribosides and examined them as potential PNP substrates in the phosphate buffer, with results summarized in Table 2. The phosphorolytic reactions were followed spectrophotometrically and, if possible, also fluorimetrically, and the results are summarized in Table 2.

**Table 2:** Kinetic parameters for enzymatic phosphorolysis of selected ribosides in 25 mM phosphate buffer, pH 6.5, at 25 °C, catalyzed by various forms of PNP. Errors about 15%.

*Substrate*	*Enzyme (wt = Wild Type)*	*K_m_ (µM)*	V_max_ (Relative) *
N7-β-d-ribosyl-Gua **	calf PNP-wt	27	0.6
N7-β-d-ribosyl-Gua **	*E. coli*-wt	~450	33
N7-ribosyl-8-azaGua	calf PNP-wt	nd	>0.13
N7-ribosyl-8-azaGua	calf PNP-N243D	nd	>1.5
N7-ribosyl-8-azaDaPur	calf PNP-wt	52	~20
N7-ribosyl-8-azaDaPur	calf PNP-N243D	>50	>60
N7-ribosyl-8-azaDaPur	*E. coli* PNP-wt	~80	~1.7
N7-ribosyl-8-azaDaPur	*E. coli* PNP-D204N	nd	~0.7 ***
N8-ribosyl-8-azaDaPur	*E. coli* PNP-wt	7	1.1
N8-ribosyl-8-azaDaPur	*E. coli* PNP-D204N	nd	~1.6 ***
N9-ribosyl-8-azaDaPur	*E. coli* PNP-wt	~20	~0.02

* relative to guanosine phosphorolysis under the same conditions (=100); ** data from ref. [1]; *** rate with 40 µM substrate.

8-Azaguanosine was reported to be a very weak substrate for mammalian PNP [11]. Similarly, only traces of activity of the calf PNP towards N7-riboside were detected. One possible reason can be unfavorable equilibrium of the phosphorolytic process, which for N9-riboside was estimated to be ~300 in favor of nucleoside synthesis, compared to ~50 for natural purines [11]. The mutated form of the calf enzyme is somewhat more active (Table 2).

As mentioned earlier, there is a considerable specificity in the phosphorolytic pathway in both calf and *E. coli* PNP in relation to 8-azaDaPur ribosides [15]. There was no detectable activity towards N9-riboside with the calf PNP, and only residual with the *E. coli* enzyme. By contrast, the N8-riboside is quite effectively phosphorolysed by the wild type *E. coli* PNP, but not by the mutant (see Table 2). Worth noting is low K_m_ value for this process (Table 2), contrasting with very high K_m_ observed in the synthetic reaction (see Table 1).

The N7-riboside of 8-azaDaPur seems to be quite rapidly and specifically phosphorolysed by calf PNP [15]. The mutation at Asn243 markedly accelerates this process (see Table 2), while virtually no activity towards N8- or N9-ribosides was detected using both wild and mutated types of PNP. This finding is somewhat surprising in view of the fact that N8-ribosides are produced in the reverse (synthetic) reaction catalyzed by wild (but not the mutated) form of calf PNP (see previous sections).

Although the spectrum of non-typical substrates of mammalian, and especially bacterial forms of PNP is broad [1,9,11], recent years brought much more examples of applications of these enzymes to synthesis and phosphorolysis of biologically interesting nucleoside analogs [10,27,28,29,30]. But the exact prediction of the substrate preferences of various forms is at present difficult [31], we think that large variability of natural PNP and possibility of mutations in the active site can offer new potentially useful catalyst for synthetic procedures in nucleoside chemistry.

### 2.3. Fluorescence of 8-Azaguanine and 2,6-Diamino-8-azapurine Ribosides and Potential Applications

Fluorescent nucleoside analogues are widely studied because of potential applications in enzymology [11,32,33]. It has been reported that many 8-azapurines and their nucleosides exhibit measurable fluorescence in aqueous medium at room temperature, which was in several instances applied to mechanistic and analytical studies [11,34,35]. The highest yields of fluorescence are observed in 8-azaxanthine and 2,6-diamino-8-azapurine, as well as in some N-alkyl derivatives of these [11,36]. Nucleosides of adenine and hypoxanthine are more fluorescent than the parent bases [13], and N8-ribosides of many 8-azapurines, including N8-ribosyl-8-azaguanine and N8-ribosyl-8-azaDaPur, exhibit yields comparable to the best fluorophores known [11].

#### 2.3.1. Fluorescence of 2,6-Diamino-8-azapurine Ribosides

Ribosylation of 2,6-diamino-8-azapurine leads to at least three fluorescent ribosides (Table 3). The N9-riboside is very highly fluorescent, but its fluorescence is generally similar, in terms of λ_max_ and decay time, to that of the free base [11]. By contrast, fluorescence of N7- and N8-ribosides is red-shifted by ~70 nm and thanks to this shift their cleavage via phosphorolysis (or hydrolysis) leads to a high fluorogenic effect at ~360 nm, which can be analytically useful (see next section).

**Table 3:** Ionization constants (pK_a_ values) and spectral parameters for neutral and ionic forms of the selected 8-azapurine ribosides and free bases. The UV spectral data are compiled from the literature [11,18,20] and fluorescence parameters determined in this and previous [11,15] works.

*Compound*	*pK_a_*	Form * (pH)	*UV Absorption*		*Fluorescence*		
			*λ_max_ (nm)*	*ε_max_ (M^−1^·cm^−1^)*	*λ_max_ (nm)*	*ϕ*	*τ (ns)*
9-β-d-ribofuranosyl-8-azaDaPur	2.9	n (7)	285	10,800	368	0.9	6
		c (2)	283	8100	360	nd **	nd **
8-β-d-ribofuranosyl-8-azaDaPur	4.9	n (7)	313	8200	430	0.41	10.6
		c (2.7)	264	13,200	430	nd **	nd **
7-β-d-ribofuranosyl-8-azaDaPur	3.95	n (7)	314	~5500	420	0.063	1.5; 0.45
		c (2)	258	~12,000	420	nd **	nd **
8-azaDaPur (free base)	3.7; 7.7	n (6)	280	8500	365	0.40	7.5; 0.2
9-β-d-ribofuranosyl-8-azaGua	8.05	n (5)	256	12,900	347	~0.01	~0.1
		ma (10)	278	11,700	362	0.55	5.6
7-β-d-ribofuranosyl-8-azaGua	7.4	n (5)	302	4900	410	~0.04	nd **
		ma (10)	304	5100	420	~0.03	nd **
8-azaGua (free base)	6.5	n (4.5)	249	11,200	395	0.05–0.33	6.2

* n—neutral form; c—cation; ma—monoanion; ** nd—not determined.

It is of interest that protonation of N7- and N8-ribosides, which leads to a significant blue shift in the UV spectra (Table 3), apparently does not alter the observed fluorescence band. This is undoubtedly due to a large shift in the acid-base equilibrium in the excided state (pK*), as compared to ground-state (pK_a_), so upon excitation the protonated 8-azapurine moiety undergoes rapid deprotonation. This process is observed also in acidified alcohols, where dual fluorescence can be observed, especially for the N8-riboside (data not shown), an analogous effect reported earlier for the N8-methyl derivative [36].

It must be stressed that the strong fluorescence of 8-azaDaPur and its ribosides is sensitive to buffer concentration, isotope exchange and other environmental factors [36], partially related to excited-state proton transfer reactions and relatively long fluorescence decay times (Table 3). This creates some difficulty in analytical applications, which can be overcome by using internal concentration standards (e.g., purified products of enzymatic reactions at standardized concentrations).

#### 2.3.2. Blood PNP Determination Using Fluorogenic Ribosides

**Figure 3:**
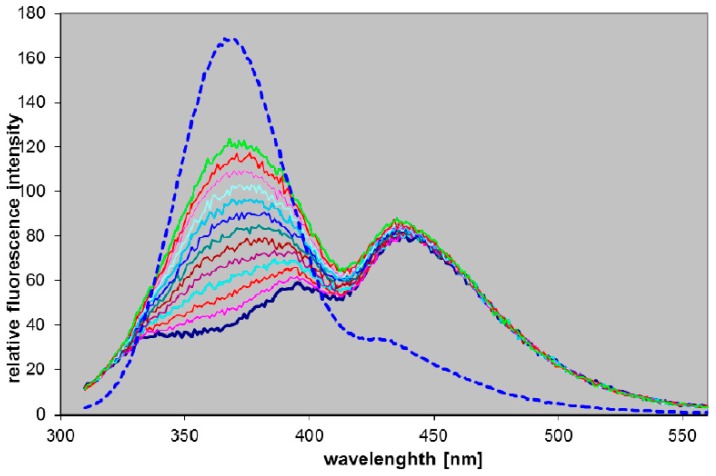
Fluorescence changes during the incubation of 16 µM N7-ribosyl-8-azaDaPur in 1000-diluted blood in the presence of 25 mM phosphate. Spectra, excited at 300 nm, were recorded every 5 min, and after 60 min an aliquot of the purified calf PNP was added, and fluorescence recorded after next 3 min (dotted line, 5-fold diminished relative to remaining curves). Note that the first (lowest) curve reflects blood fluorescence background, and minimum at 415 nm is due to the re-absorption by hemoglobin.

It has been reported earlier that 7-β-d-ribofuranosyl-8-azaDaPur is a highly fluorogenic substrate for calf PNP in phosphate buffer [15]. Human PNP is similar to the calf enzyme [1], and we have therefore verified a possibility to detect PNP activity in human blood using the same substrate. The experiment was run with 1000-fold diluted, lysed human blood in 25 mM phosphate, and in a phosphate-free 20 mM HEPS buffer, pH 6.6, as a control. *Ca.* 16 µM N7-riboside was used as a substrate. Virtually no fluorescence change was observed in the phosphate-free buffer (data not shown), but in the presence of phosphate the appearance of an emission band at 365 nm indicated free base production (Figure 3, Table 3).

The apparent K_m_ for this process was rather high, ~90 µM (data not shown), and the presented fluorogenic effect (Figure 3) can undoubtedly be enhanced by optimizing assay conditions. Although phosphorolysis of 7-β-d-ribofuranosyl-8-azaDaPur is slow in comparison to the best PNP substrates, like guanosine or 7-methylguanosine [11], the fluorogenic effect is large thanks to high fluorescence yield of the product (~0.4), and the present assay, in its optimized version, may be more sensitive and simpler than other assays proposed for this enzyme [1,37].

Of special interest is high selectivity of the fluorogenic ribosides of 8-azaDaPur to various forms of PNP, namely, mammalian (N7-ribosides) and bacterial (N8-ribosides), particularly in view of recent applications of bacterial (*E. coli*) PNP as a suicidal-gene in cancer chemotherapy [8]. These two forms of PNP can likely be assayed selectively in the same blood sample, with no pre-purification necessary.

## 3. Experimental Section

Recombinant *E. coli* and calf spleen PNP, as well as mutated form of these, were obtained and purified as described elsewhere [17,38]. Stock solutions (0.1–1.7 mM per subunit) were stored frozen at −20 °C and diluted prior to experiments. 2,6-Diamino-8-azapurine sulfate, N7-methylguanosine (m^7^Guo) and 8-azaguanine were from Sigma-Aldrich (St. Louis, MO, USA), the latter was re-crystallized as a monosodium salt. N7-methylguanosine was used without further purification.

Fluorescence was measured on a Varian Eclipse instrument (Varian Corp., Palo Alto, CA, USA), and UV absorption kinetic experiments on a Cary 300 (Varian). All buffers were of analytical grade and displayed no fluorescence background.

α-d-Ribose-1-phosphate (100 mM solution in 100 mM HEPES buffer, pH ~7.2) was prepared enzymatically from the N7-methylguanosine and inorganic phosphate, using the modified procedure of Krenitsky *et al.* [39]. The recombinant calf PNP (~2 µg per 1 mL reaction volume) was used as catalyst, and the reaction progress was monitored fluorimetrically [40]. Alternatively, the N243D mutant can be used as a more selective towards m^7^Guo [41]. The second reaction product, N7-methylguanine, was removed in nearly 97% by spontaneous crystallization and filtration. The phosphorylated ribose solution was stored at −20 °C and assayed using previously described fluorimetric method [37]. It was found that 1-year storage caused hydrolysis of not more than 20% of the compound.

Synthetic reactions were carried out on a milligram scale as previously described [14], typically in 1 mL volume, in HEPES buffer. Enzyme concentrations were 1–10 µg/mL, and ribose-1-phosphate ~5 mM. After 24 h reaction mixtures were frozen.

Reaction products were analyzed by the analytical reverse-phase HPLC on a UFLC system from Shimadzu (Kyoto, Japan) equipped with UV (diode-array) detection at 280 nm and 315 nm. The column used was a Kromasil reversed-phase analytical C8 column (250 × 4.6 mm, 5-μm particle size). For product separation, an analogous semi-preparative column was used. The eluent was deionized water (10 min), followed by a linear gradient from 0% to 30% methanol (60 min). The reactions were carried out at pH ~6.5, and samples containing 8-azaguanine ribosides were acidified to ~5 prior to the HPLC analysis.

Kinetic parameters of the enzymatic reactions were calculated using linear regression analysis of the double-reciprocal plots. Substrate concentration in the kinetic experiments ranged typically from 1 to ~200 µM, and enzyme concentrations were adjusted so that the reaction rates were in the range 0.1 to ~5 uM/min. Fluorescence measurements were conducted in semi-micro cuvettes (pathlength 0.4 cm, volumen 1 mL) to diminish the inner filter effect.

## Abbreviations

PNP, purine-nucleoside phoshorylase; 8-azaGua, 8-azaguanine; 8-azaDaPur, 2,6-diamino-8-azapurine.
